# Serious gaming as potential training tool for recognition of adverse drug reactions: side-effect exposure—medical education (*SeeMe*)

**DOI:** 10.1007/s00228-024-03739-w

**Published:** 2024-08-19

**Authors:** Ingmar Bergs, Laura Bell, Sebastian Fedrowitz, Tim Krüger, Martin Lemos, Julia C. Stingl, Katja S. Just

**Affiliations:** 1 Institute of Clinical Pharmacology, RWTH Aachen University Hospital, Pauwelsstr. 30, 52074 Aachen, Germany; 2 Audiovisual Media Center, Medical Faculty, RWTH Aachen University, Aachen, Germany; 3 Department of Pneumology and Internal Intensive Care Medicine, RWTH Aachen University Hospital, Aachen, Germany

**Keywords:** Medical education, Pharmacogenetics, Serious gaming, Adverse drug reactions, UPGx

## Abstract

**Purpose:**

The recognition of adverse drug reactions (ADRs) is an important part of daily clinical work. However, medical education in this field is mostly drug-based and does not address adequately the complexity of this field regarding individual risk factors and polypharmacy. This study investigates the potential of the web-based serious game *SeeMe* (side-effect exposure—medical education) in pharmacological education of medical students to improve the recognition of relevant ADRs.

**Methods:**

One hundred fifty-seven medical students were recruited to evaluate the serious game *SeeMe*. *SeeMe* was developed to improve knowledge and recognition of ADRs in clinical practice. Players take on the role of a physician trying to understand fictional patients with ADRs. Before and after an 8-week playing period, an evaluation was carried out through a pre- and post-questionnaire and a pre- and post- knowledge test.

**Results:**

The students achieved significantly better results in the knowledge test, as almost twice as many exam-relevant questions were answered correctly (*p* < 0.001). The serious game had a positive effect on the students’ perception of the importance of ADRs.

**Conclusion:**

This study demonstrates the potential of web- and case-based fictional serious games in medical education. The improved recognition of side effects represents a crucial step for education and training in clinical pharmacology. Future versions of the serious game may take this further and focus on training in the treatment of ADRs and their relevance in various healthcare professions.

**Supplementary Information:**

The online version contains supplementary material available at 10.1007/s00228-024-03739-w.

## Introduction

Recognizing adverse drug reactions (ADR) in everyday clinical practice is a challenge for medical disciplines because ADRs can present themselves differently in each individual patient [1]. Next to patient-specific risk factors like age or comorbidities, polypharmacy and pharmacogenetic (PGx) aspects play a role [2–4]. ADRs are clinically highly relevant and approximately 6.5% of all admissions to the emergency department can be related to an ADR [5–7]. However, due to its various manifestations, ADRs often remain undetected [5]. This is a critical challenge for our healthcare system, as pharmacovigilance research relies on the detection and reporting of ADRs to improve patient safety in drug therapy [8].

Teaching pharmacological knowledge and skills in relation to ADRs is a challenge in studies of medicine and is usually based on books or tables listing the known side effects associated with the use of certain drugs. A literature review concluded that there is an urgent need to improve education in pharmacovigilance [9]. And 5 years ago, a stakeholder meeting organized by the Netherlands Pharmacovigilance Centre Lareb on behalf of the World Health Organization (WHO) even introduced a core curriculum for teaching drug safety aspects at universities [10].

In the context of increasing digitalization, more and more opportunities have arisen in recent years to establish digital learning formats within traditional medical education. Particularly, the SARS-CoV2 pandemic has shown how urgently new learning formats are needed to maintain practical training in the medical field [11, 12]. Serious games are defined as digital games designed for purposes beyond mere entertainment, aiming to educate, train, and solve problems in various fields, including medical education. A serious game-based approach can provide a safe and secure introduction to patient contact and reinforce theoretical content. It may be at least as effective as traditional training and even superior in terms of improving knowledge, skills, and satisfaction [13]. For example, a serious game for primary healthcare education showed to be just as effective as studying through books [14]. A recent review concludes that serious games are, and game-based learning is becoming more popular in medical education due to its innovative, interactive methods that enhance student learning experiences and improve outcomes [15]. Such learning formats offer the opportunity to deliver learning content in a more individual and hands-on way, regardless of the time spent. There is an increased demand for learning formats such as serious games in education, particularly among pharmacy students [16–18]. However, to the best of our knowledge, there is currently no serious game available that allows the training of ADR recognition.

The current study investigated whether a newly developed serious game called *SeeMe* (side-effect exposure—medical education) can be used to train medical students’ recognition of ADRs in clinical pharmacology.

## Methods

### Serious game development

The development of the serious game *SeeMe* was part of the European U-PGx project (European Union’s Horizon 2020 research and innovation program, grant agreement no. 668353) and the Exploratory Teaching Space program of the RWTH Aachen University (Germany). Within the framework of a European-wide cooperation, the U-PGx project tested the use of PGx guided treatment for the reduction of ADRs [4]. The development of the game was part of the education and training program developed for successful implementation of PGx-guided treatment into clinical practice. The game is accessible at https://upgx.eu/category/documents/.

The game was designed and developed in-house by the Audiovisual Media Center of the Medical Faculty, RWTH Aachen University, in close cooperation with the Institute of Clinical Pharmacology and is available to medical students from their third year onwards at RWTH Aachen University.

**Fig. 1:**
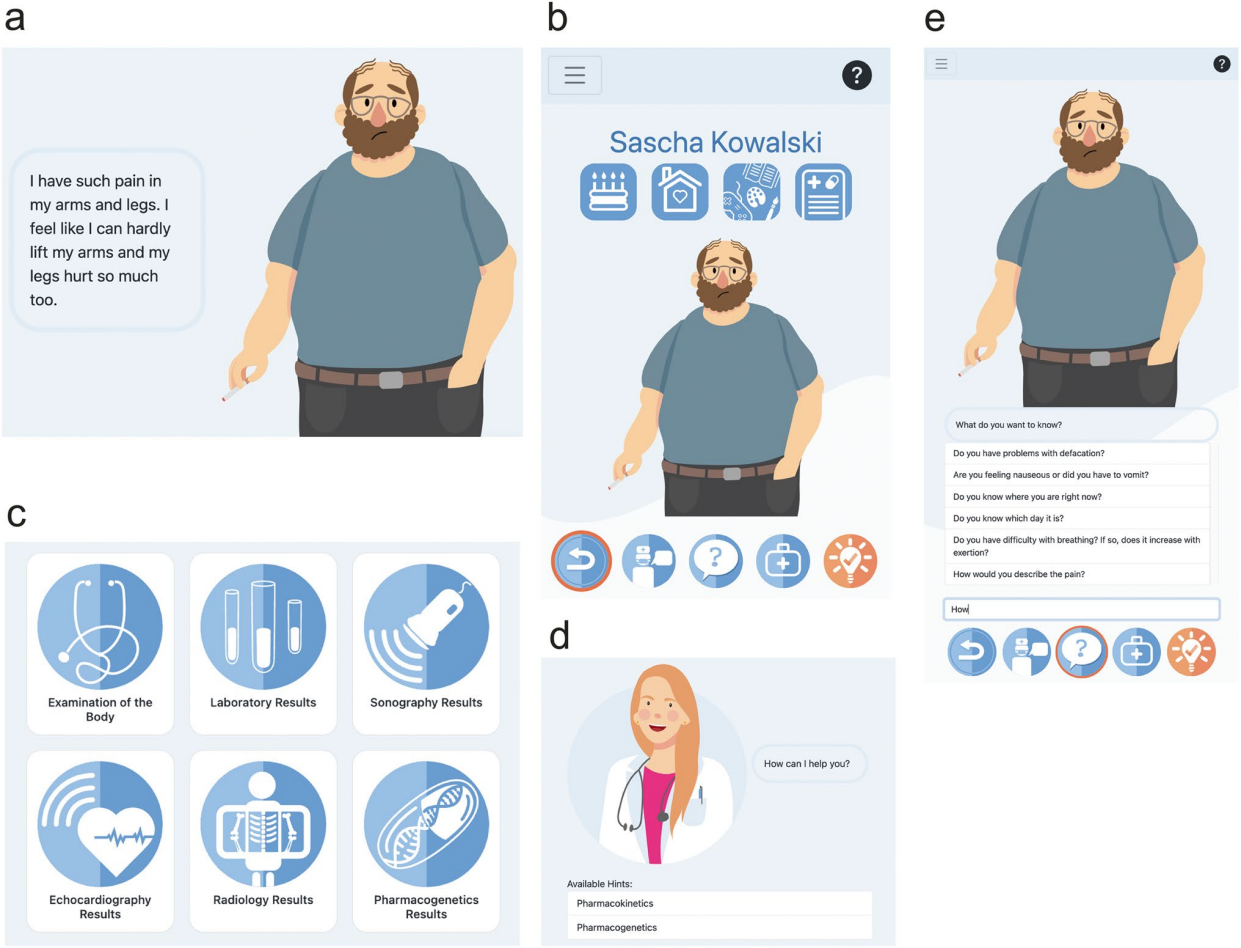
**a** Within the introduction, the patient Sascha Kowalski talks about his complaints. **b** The game interface. **c** Students can choose between several examinations. **d** When stuck, students can ask for help and tips from a doctor. **e** Students can ask Sascha Kowalski about his complaints and ask various questions

*SeeMe* is a web- and case-based serious game in which the player takes on the role of a physician and faces 47 patient cases. Fictional patients can present with a wide variety of complaints that can be related to a relevant ADR of a previous drug therapy. The emphasis is put on the recognition of ADRs in a patient with different drugs and comorbidities rather than the enumeration of side effects linked to drugs such as presented in pharmacological courses or books. All cases reflect clinically relevant and common ADRs, based on questions frequently asked in the final state exams in Germany and are graphically illustrated. For an extensive overview of all 47 cases see Supplementary Table S1. A short impression of the game interface and one of a fictional patient is given in Fig. 1a, b. The player must arrive at a diagnosis, think about potential drug treatments for that diagnosis and identify the ADR by independently asking about complaints (Fig. 1e) and by assessing medical findings such as a physical examination, laboratory values, imaging, or PGx data (Fig. 1c). During the game, players can request help and tips from a virtual expert (Fig. 1d). At the end of each patient case, the player must answer whether the symptoms of the current patient are mainly due to ADRs, and which diagnoses should be made. In addition, two 5-point Likert scales assess whether the players rate the current patient case as easy and realistic. After confirming the answers, players receive feedback, and, if they wish to complete the case (in which case they cannot replay the current case), they can access the solution with an explanation of the case and the corresponding ADR.

### Study population

The evaluation of *SeeMe* was conducted monocentric at the Medical Faculty of the RWTH Aachen University, Germany. For this purpose, medical students were recruited who were already in clinical and practical training within their studies and had patient contact starting from the third year onwards. Students participated voluntarily as part of a course, and informed consent was obtained from all participants. The study period was from 07/2021 to 02/2024 and comprised three study years. The study was conducted in digital form and participation was anonymous, with an anonymized identifier used to link online questionnaires and game data. The study was performed in line with the principles of the Declaration of Helsinki. Approval was granted by the local ethical committee (EK 230/21) of the medical faculty of RWTH Aachen University.

### Game evaluation, knowledge tests, and study course

Before the study, a short lecture was given on the relevance of ADRs, and the serious game was introduced. An anonymous online survey was conducted asking students’ attitudes and self-assessment towards pharmacology, study progress, and the importance of ADRs. The survey was presented through the online platform LimeSurvey [19]. Items needed to be rated on a 5-point Likert scale ranging from 1 “don’t agree at all” to 5 “totally agree.” In addition, the students were tested on their knowledge of ADRs using an online formative test. The knowledge test was adapted to the examination mode of German state examinations, using 30 multiple choice questions (Supplementary Table S2). The questions were designed in pairs to cover comparable topics of drug-ADR combinations in pre- and post-serious game knowledge tests. The questions were prepared by two physicians under a dual control principle and supervised by a professor of clinical pharmacology. This was done to ensure comparable levels of severity in both exams, before and after game play. Due to the formative nature of the knowledge test, there was an option to answer with “I don’t know.” Students were informed that this test had no impact on course grades.

Following the pre-evaluation, students had 8 weeks to play the serious game. They were required to play all cases and were allowed to repeat cases as many times as they liked. Subsequently, participants were surveyed again with an online questionnaire. The post-questionnaire assessed game experience and usability, as well as the subjective learning progress. Usability was assessed using the System Usability Scale (SUS) [20, 21]. The SUS is a ten-item scale that provides a single number, ranging from 0 to 100, representing a composite measure of overall usability. In addition, students’ knowledge was assessed a second time. The second knowledge test contained the same subject as before and was not changed in severity but was adjusted in language and logical linkage.

### Statistical analysis

Statistical analyses were performed in R [22]. For all analyses, the level of significance was set at *α* = 0.05. Epidemiological data of the participants were collected and reported in absolute values and percentages and were appropriate with mean (M) and standard deviation (SD). To test whether students’ opinions changed and whether they were able to increase their knowledge, pre- and post- questionnaires, and knowledge tests were compared. Because of a skewed distribution and lack of homogeneity of variance for several items, Mann–Whitney *U* tests were applied for pre- and post-comparison using the R package *stats*. The *rcompanion* package was used to calculate the effect size *r*. To test the potential effect of study year and gender on knowledge and the potential interaction between study year, gender, and knowledge gain in the pre- and post- exam, a linear mixed-effects model with time (pre, post) as within-subject factor and study year (3rd to 5th) and gender (female, male, divers) as between-subject factors was conducted using the *nlme* package. Further, the MuMIn package was used to provide the marginal and conditional R. The *ggplot2* and *ggstatsplot* packages were utilized for data visualization.

## Results

### Study cohort

In total, *N* = 157 students enrolled and completed the serious game course. Of them, 77.7% (*n* = 122) were female and 22.3% (*n* = 35) were male. Characteristics of the cohort are summarized in Table 1.

**Table 1:** Basic characteristics of the participants of the current study

	**Total** **∑ *n* = 157**
Male, *n* (%)	35 (22.3)
Female, *n* (%)	122 (77.7)
Age (years), *n* (%)	
< 20 years	4 (2.5)
20–25 years	127 (80.9)
26–30 years	18 (11.5)
> 30 years	8 (5.1)
Study year, *n* (%)	
3rd	66 (42)
4th	67 (42.7)
5th	23 (14.7)
NA	1 (0.6)

All numbers rounded to integral numbers

Around a third of students (30%, *n* = 46) indicated that they use books as their primary learning medium, and 21% (*n* = 33), respective 17% (*n* = 26) that they already used apps or videos as a learning medium. Although only some participants (*n* = 31) play online games in their leisure time, participants stated pre-game experience that they were open to online learning games to deepen and consolidate their knowledge (mean 4.8, SD 0.6).

### Evaluation: pre- and post-gaming

There was high agreement of a mean 4.2 (SD 0.8) on a 5-point Likert scale, that ADRs were realistic and transferable to clinic. The usability of the serious game was rated as good, with a SUS mean of 77.7 (SD 15.3). See Table 2 for an overview of the self-assessments pre- and post-serious game play.

**Table 2:** Results of the pre- and post-evaluation

Question	Pre, mean (SD)	Post, mean (SD)	*n*	*p*-value
**I feel confident in handling and prescribing medications**	1.8 (0.8)	2.4 (0.9)	155	< 0.001
**I am afraid of harming my patient by prescribing a medication**	3.8 (0.9)	3.5 (0.9)	148	0.001
**I consider my knowledge level regarding pharmacology and the recognition of side effects to be improved after using the serious game**	-	3.8 (0.9)	152	-
**I would like more professional support and training in the field of pharmacology and medicine**	4.4 (0.7)	4.3 (0.7)	139	0.02
**The online game has positively improved my perception of the issue of adverse drug reactions**	-	4.2 (1.0)	157	-

All items ranked anonymously on a 5-point Likert scale from 1 “don’t agree at all” to 5 “agree totally.” Item 3 was only assessed once after using the serious game. *SD* standard deviation.

Confidence to handle medications and to prescribe them was low across time (pre-, post-game play) but still improved significantly after using *SeeMe* from mean 1.8 (SD 0.8) to 2.4 (SD 0.9), *V* = 790.5, *p* < 0.001, *r* =  − 0.36. Along with this, a certain anxiety to harm patients with prescribing a medication reduced slightly, yet significantly from 3.8 (SD 0.9) to 3.5 (SD 0.9), *V* = 3078, *p* = 0.001, *r* = 0.14. The wish for more support and training in the field of pharmacology was underlined by participants with an overall agreement of a mean of 4.4 (SD 0.7) before and 4.3 (SD 0.7) after playing the game (*V* = 1231.5, *p* = 0.02, *r* = 0.12). Students stated that their knowledge improved modestly after using the serious game with a mean 3.8 (SD 0.9), although it was still considered to be low. Yet, students highly agreed that the use of the serious game made the topic more accessible to them (mean 4.2, SD 1.0).

### Exam results

**Fig. 2:**
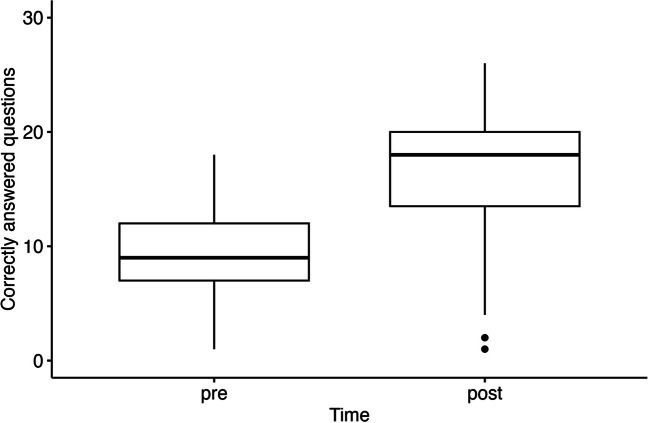
Boxplots showing correctly answered questions of the exam before and after using the serious game

Due to a technical error, the exam results were only saved for the years 2021 to 2022 (*n* = 110). Test results in the formative exam during these years improved significantly from 31% (9.4, SD 4.9) correct answers before playing the game to 55% (16.6, SD 5.1) correct answers after game play (Fig. 2**)**, i.e., a significant main effect of time (pre, post) was found, *F*(1,98) = 324.97, *p* < 0.001. Notably, the marginal *R*^2^ for the fixed effects was 0.56, and the conditional *R*^2^ for both fixed and random effects was 0.75. Further, there was a significant effect of study year, and students in higher semesters reached better exam results, *F*(5,98) = 14.63, *p* < 0.001. However, the interaction between study year and time (pre-, post-game play) was not significant, indicating that the year of study did not influence the increase in knowledge, *F*(5,98) = 1.25, *p* = 0.29. Similarly, no significant gender differences were observed in exam results, *F*(1,98) = 0.55, *p* = 0.46.

### Game data

More than half of the students, 56.1% (*n* = 88) played the game at least twice a week. Students started 16,939 times a patient case, of which 98.1% (*n* = 16,615) patient cases were finished. Each student finished between 30 and 47 cases. On average, in 63.6% (*n* = 7408) of game sessions, students agreed that patient cases were realistic, and in only 31.2% (*n* = 3652) of game sessions, students agreed that patient cases were easy. Students used the options to ask questions to the patient between 0 and 27 times and requested help from the fictional doctor within the game between 0 to 42 times. In 70% (*n* = 11,865 out of 16,939) of game sessions, students identified the presence or absence of ADRs correctly.

## Discussion

In this study, we reversed the conventional learning method and concentrated on recognizing ADRs in patients, as well as identifying the probable drugs that may have typically caused these ADRs, within the context of a serious pharmacology game *SeeMe*. The current evaluation of *SeeMe* showed a marked improvement in the recognition of ADRs in medical students. Students were able to significantly improve their exam-relevant knowledge by answering almost twice as many questions correctly. Generally, the serious game was rated positively by most students, particularly regarding the learning effect, and was classified as realistic and transferable to clinical practice. While several studies have already shown that serious games are equivalent to traditional learning formats and can improve knowledge [13, 23], to our knowledge, this is the first published study on a serious game to improve ADR recognition in medical students. Notably, although the serious game increased students’ confidence in prescribing medication and reduced their fear of harming patients, confidence remained low. This is reasonable, as students still need a lot of training to enable working as physicians, as here students starting from the 3rd year of training were enrolled. In addition, treatment was only indirectly trained through the correct recognition of ADRs. This emphasises the need for increased and sufficient training of medical students in relation to ADRs.

Personalized medicine using PGx promised to improve drug efficacy and drug safety [24, 25]. The European Ubiquitous Pharmacogenomics (U-PGx) project showed that the use of a 12-gene PGx panel along with treatment recommendations can reduce the occurrence of ADRs by 30% [4]. However, relevant barriers and challenges to the implementation of PGx in clinical work include a targeted education and its useful implementation [26, 27]. A symptom-based approach such as the current serious game is considered a good tool to better integrate existing knowledge about PGx and the resulting clinically relevant ADRs. Training in the recognition of ADRs in everyday clinical practice is particularly important to avoid harming patients [28]. Education and training, including the development of the here presented serious game, were therefore part of the U-PGx project. Often a drug-based approach needs to be taken to identify potential ADRs, whereas in clinical reality, the focus is primarily on a symptom that needs to be associated with a drug. However, the manifestation of an ADR depends on numerous individual factors and can present variably in clinical practice [1]. As an example, there are many drugs causing agranulocytosis a potentially life-threatening ADR [29]. Thus, besides knowing facts and reproducing them in a medical term in an exam, recognition in clinical work is most crucial and should be trained to improve drug safety.

The results presented should be interpreted considering some limitations and recommendations for future follow-up studies should be considered. First, knowledge tests were carried out directly after playing the game. This does not allow any conclusions to be drawn about long-term learning effects. Future studies should carry out knowledge tests later and possibly evaluate the application of the acquired knowledge in practice. In addition, the effect of certain knowledge questions or topics might be addressed in a follow-up study. Due to a technical error, only the overall knowledge test results could be retrieved in the current study. Further, students also attended other lessons and training sessions in addition to the serious game. It therefore cannot be completely ruled out that at least part of the learning success can be attributed to learning outside the serious game. In this context, however, it should be noted that the results of the study were comparable across and independent of the study years, and since the knowledge tests were formative, no learning was provoked by the study design.

Future studies might investigate the game play in more detail. In the current study, players were allowed to take as much time as desired to finish a patient case. As it was also possible to take longer breaks during a patient’s game, the actual playing time cannot be tracked and compared between patient cases and students at present. Similarly, patients without ADRs as well as the treatment of patient could be added to provide a more realistic setting and address the training aspect in more detail. Furthermore, the expansion of the application in the training of other healthcare professions, which are also related to the topic of ADRs and their recognition or patients, should be the subject of further investigation. Nursing and pharmaceutical professions could also benefit from using the game. A second version of the game is currently being developed that considers treatment of patients, patients without ADRs, other professions, and factors such as the seriousness and urgency of a case, which can be addressed in the game.

## Conclusion

Overall, the study indicates that the implementation of new digital learning tools in pharmacology training, specifically in pharmacovigilance, can be successful and can improve knowledge in the field of ADRs. It offers an additional tool to the existing training formats and may in future potentially even provide an EU-wide training platform for the recognition and treatment of ADRs.

## Supplementary Information

Below is the link to the electronic supplementary material.

Supplementary file1 (DOCX 85 KB)

## Data Availability

The datasets generated during the current study are available from the corresponding author on reasonable request.
