# Supplementary material for: Serious gaming as potential training tool for recognition of adverse drug reactions: side-effect exposure—medical education (SeeMe)
Source: Eur J Clin Pharmacol. 2024 Aug 19;80(11):1787–93. doi: 10.1007/s00228-024-03739-w (PMC11458730; doi:10.1007/s00228-024-03739-w)
Supplement: Supplementary file 1 — Supplementary file1 (DOCX 85 KB) [file 228_2024_3739_MOESM1_ESM.docx]

**Supplement S1:** Overview of cases of the *SeeMe* serious game.

| **Case character** | **Case number** | **Case description** | **Reason for consultation** | **Pharmacogenetic content** |
| --- | --- | --- | --- | --- |
| Female, 19 years old | 1 | Leg vein thrombosis under contraception | Swelling of the right leg | - |
|  | 2 | Attempted suicide | Suicide attempt | - |
|  | 3 | Out-of-control INR under phenprocoumon | Bilateral pulmonary embolism | ✓ |
| Female, 32 years old | 1 | Seizure under bupropion | Seizure | ✓ |
| Female, 80 years old | 1 | Bleeding under full anticoagulation | Blood in stool | - |
|  | 2 | Dark black stool | Dark black stool | - |
|  | 3 | Confusion after outpatient endoscopy | Confusion after colonoscopy | - |
| Female, 24 years old | 1 | Blood count changes under dapsone | Increasing headache, shortness of breath, and lethargy | ✓ |
|  | 2 | Agranulocytosis under metamizole | Increasing discomfort and fever | - |
| Male, 54 years old | 1 | Hyperthyroidism | Restlessness and increasing sweating | - |
|  | 2 | Hearing disorder under diuretics | Deafness | - |
|  | 3 | Vitamin B12 deficiency under PPI and metformin | Fatigue and lack of drive | - |
|  | 4 | Urinary tract infection under SGLT2 inhibitors | Painful urination and fever | - |
| Female, 49 years old | 1 | Skin rash under carbimazole and doxepine | Skin rash | - |
|  | 2 | Kidney failure under complementary slimming capsules | Worsening condition | - |
|  | 3 | Renal transplant rejection despite medication with tacrolimus | Fatigue after kidney transplantation | ✓ |
| Female, 42 years old | 1 | Acute adrenal insufficiency after immediate stop of predisone | Fatigue and discomfort | - |
| Male, 49 years old | 1 | Carbamazepine-induced Stevens-Johnson syndrome | Fatigue, sore throat, fever, and painful skin detachment | ✓ |
| Male, 48 years old | 1 | Reinfarction under antiplatelet medication | New onset left thoracic chest pain | ✓ |
|  | 2 | Drug exanthema under antibiotic treatment | Skin rash | - |
| Female, 64 years old | 1 | Cardiomyopathies after chemotherapy | Shortness of breath with minimal exertion | - |
|  | 2 | Necrosis of the jaw under bisphosphonate | Pain and open wound in the mouth | - |
| Male, 64 years old | 1 | QT time prolongation under multi-medication | Heart palpitations | - |
|  | 2 | Irritable cough under ACE inhibitors | Worsening cough | - |
|  | 3 | Syncope under sildenafil | Syncope | - |
|  | 4 | Nausea and diarrhoea under phosphodiesterase-4 inhibitor | Nausea and diarrhea | - |
| Male, 42 years old | 1 | Statin-associated myopathy | Muscle pain | ✓ |
|  | 2 | Ankle oedema under calcium antagonists | Ankle edema | - |
| Female, 52 years old | 1 | Hand-foot syndrome under chemotherapy | Redness and blisters on hands | - |
|  | 2 | Polyneuropathy after oxaliplatin | Tingling in hands and feet | - |
| Male, 72 years old | 1 | Diarrhoea under antifibrotic medication | Persistent diarrhea | - |
|  | 2 | Tardive dyskinesia under antipsychotics | Tics | - |
| Female, 88 years old | 1 | Unspecific symptoms of fatigue and agitation under multi-medication | Confusion under polypharmacy | ✓ |
| Male, 28 years old | 1 | Psychosis after trip to Africa | Psychotic experience | - |
|  | 2 | Achilles tendon rupture after urinary tract infection | Achilles tendon rupture after urinary tract infection | - |
|  | 3 | Drug-induced headache | Persistent headache | - |
| Female, 27 years old | 1 | Malignant neuroleptic syndrome under comedication | Muscle stiffness, parkinsonian movement, increased temperature, restlessness | - |
| Female, 73 years old | 1 | Intoxication with codeine as an antitussive | Increasing drowsiness and shallow breathing | ✓ |
| Male, 72 years old | 1 | Pathological gamble behaviour under dopamine agonists | Gambling addiction | - |
|  | 2 | Fracture under PPI | Fall | - |
| Male, 32 years old | 1 | Oral candidiasis under comedication | Bad breath, furry feeling in mouth, and pain when swallowing | - |
|  | 2 | Nasal spray dependence | Permanently swollen nose and poor smell | - |
| Female, 22 years old | 1 | Nausea and vomiting under treatment of tuberculosis | Nausea and repeated vomiting | - |
|  | 2 | Pulmonary embolism despite full anticoagulation | Increasing shortness of breath for 2 days | - |
| Male, 24 years old | 1 | Weight gain under antipsychotic treatment | Psychotic experience | - |
|  | 2 | Acute dystonia under antipsychotic treatment | Suspected seizure | - |

As the first version of the serious game *SeeMe* was developed as part of the European Ubiquitous Pharmacogenomics project, some cases include pharmacogenetic variants. Despite pharmacogenetic variants, many individual factors such as age, sex, pharmacodynamic, and pharmacokinetic drug-drug interactions are part of the cases, but not shown in detail in this table to keep playing the game interesting.

**Supplement S2:** Examples of the multiple-choice questions, performed in the pre and post exam

| Pre Test | Post Test |
| --- | --- |
| Agranulocytosis caused by metamizole....   1. is not dose dependent 2. occurs within 24 h 3. is associated with low lethality 4. is not associated with metamizole 5. Do not know | Agranulocytosis caused by metamizole....   1. usually occurs with some time delay 2. Is dose dependent 3. Leads to death in 80% 4. Is not associated with each other 5. Do not know |
| Which statement about anthracyclines is most likely true?   1. Chronic cardiotoxicity is dose-dependent 2. Chronic cardiotoxicity is reversible over years 3. The damage to the myocardium is due to the cytostatic effect 4. The acute toxic effects are irreversible 5. Do not know | Which statement about anthracyclines is least true?   1. Chronic cardiotoxicity is reversible over years 2. The damage to the myocardium is not due to the cytostatic effect 3. The acute toxic effects are reversible 4. A cardiac ultrasound should be performed in all patients before and after therapy 5. Do not know |
| A patient with COPD has been complaining of an increasing irritable cough for some time. In the examinations you do not find an organic cause. Which medication could most likely explain the complaints?   1. Ramipril 2. Valsartan 3. Gabapentin 4. Amlodipine 5. Do not know | A patient has been complaining of a dry, irritating cough for some time. Which discontinued antihypertensive drug is most likely to cause the discomfort?   1. Lisinopril 2. Valsartan 3. Ebrantil 4. Amlodipine 5. Do not know |
| Proton pump inhibitors are at least associated with....   1. Renal dysfunction 2. Vit. B12 deficiency 3. Liver elevation 4. Increased fracture risk 5. Do not know | Proton pump inhibitors in long-term use are most likely associated with...   1. Vit. B12 deficiency 2. Renal dysfunction 3. Myopathy 4. Hypothyroidism 5. Do not know |

Selected questions have been adapted to the learning level of the German state examination for medical students and the examination mode has been adjusted accordingly. The questions are not grouped by subject but are technical questions about the side effect and the pharmacological context. Students were allowed to quit the question by choosing “don’t know” option. The content of questions was lightly varied in pre and post testing. For ease of presentation, answer option A) is the correct answer to the question; the answers were mixed during the test.
